# Screening strategies for adults with type 2 diabetes mellitus: a systematic review protocol

**DOI:** 10.1186/s13643-020-01417-3

**Published:** 2020-07-13

**Authors:** Helen Mearns, Paul Kuodi Otiku, Mary Shelton, Tamara Kredo, Benjamin M. Kagina, Bey-Marrié Schmidt

**Affiliations:** 1 Vaccines for Africa Initiative, University of Cape Town, Cape Town, South Africa; 2 School of Public Health & Family Medicine, University of Cape Town, Cape Town, South Africa; 3 Health Sciences Library, University of Cape Town, Cape Town, South Africa; 4 Cochrane South Africa, South African Medical Research Council, Cape Town, South Africa; 5 Division of Clinical Pharmacology, Department of Medicine, Faculty of Medicine and Health Sciences, University of Stellenbosch, Cape Town, South Africa

**Keywords:** Screening, Mass screening, Targeted, Opportunistic, Type 2 diabetes mellitus

## Abstract

**Background:**

There is limited evidence on whether screening for type 2 diabetes mellitus affects health outcomes. A recent systematic review of randomised clinical trials found only one trial that met their inclusion criteria; therefore, current guidelines for screening interventions for type 2 diabetes mellitus are based on expert opinions and best practice rather than synthesised evidence. This systematic review seeks to collate evidence from non-randomised studies to investigate the effect of screening for adults with type 2 diabetes on outcomes including diabetes-related morbidity, mortality (all-cause and diabetes-related) and harms.

**Methods:**

This systematic review will follow Effective Practice and Organisation of Care (EPOC) guidelines for the synthesis of non-randomised studies. We will search PubMed/MEDLINE, Scopus, Web of Science, CINAHL, Academic Search Premier and Health Source Nursing Academic (from inception onwards). We will include non-randomised trials, controlled before-after studies, interrupted time-series studies, repeated measures studies and concurrently controlled prospective cohort studies. The primary outcome will be diabetes-related morbidity (microvascular complications of diabetic retinopathy, nephropathy or neuropathy or macrovascular complications of non-fatal myocardial infarction, peripheral arterial disease or non-fatal stroke). The secondary outcomes will be mortality (all-cause and diabetes-related) and harms of screening strategies to patients (including psychological harms or adverse events following treatments) or to health care system (including resource allocation for false-positives or overdiagnosis). Two reviewers will independently screen all citations and full-text articles. Data will be abstracted by one reviewer and checked by a second. The risk of bias of individual studies will be appraised using the ROBINS-I tool. GRADE will be used to determine the quality of the scientific evidence. If feasible, we will conduct random effects meta-analysis where appropriate. If necessary, analyses will be conducted to explore the potential sources of heterogeneity (e.g. age, sex, socio-economic status, rural versus urban or low-middle income versus high-income country). We will disseminate the findings via publications and through relevant networks.

**Discussion:**

The protocol outlines the methods for systematically reviewing and synthesising evidence of screening strategies for type 2 diabetes mellitus and their effect on health outcomes associated with the disease. The potential impact of this systematic review is improved evidence-informed decision-making for policies and practice for screening of type-2 diabetes.

**Systematic review registration:**

PROSPERO CRD42020147439

## Background

### Description of the condition

Diabetes mellitus is a disease of increasing global concern. The global prevalence of diabetes was approximately 425 million people in 2017, approximately 8.5% of the adult population, and is expected to double by 2045 [1]. In high-income countries, type 2 diabetes mellitus accounts for approximately 90% of diabetes cases; there is insufficient data to estimate the ratio of type 2 diabetes mellitus in low- and middle- income countries, but it is assumed to be similar [1, 2]. Clinical diabetes is diagnosed through the detection of elevated levels of glucose in the blood (hyperglycemia) [3]; however, it is estimated that half of the people who have diabetes are not diagnosed [1].

In addition to those individuals who have clinical diabetes, another 352 million, approximately 7.3% of the adult population, have intermediate blood glucose levels that are considered in between normal and clinically diagnosed diabetes [1, 3]. These intermediate blood glucose levels perform as a risk score, where increasing values are associated with an increasing likelihood of progression to diabetes, cardiovascular disease and all-cause mortality [2, 4]. Patients who present with intermediate levels of blood glucose are described using a number of terminologies including mild glucose intolerance, non-diabetic hyperglycaemia and prediabetes. The terminology promoted by the World Health Organization (WHO) is impaired glucose tolerance (IGT), impaired fasting glucose (IFG) and intermediate hyperglycaemia [3, 5]. The term prediabetes is gaining in popularity even though the WHO has warned its use may lead to disease stigma and detract from the significant cardiovascular risk of this population [5]. About a third of people with IGT and IFG are young, aged between 20–39 years, meaning they will spend many years at risk of developing diabetes [1]. Other risk factors, apart from intermediate glucose levels, for the development of diabetes are increasing age of more than 45 years and obesity [2].

Type 2 diabetes mellitus arises due to defective insulin activity in body tissues, defective insulin secretion from pancreas or a combination of the two [2]. Type 2 diabetes mellitus usually occurs in older adults, but with a change in lifestyle factors, such as inactivity and obesity, the condition is increasingly being detected in children, adolescents and young adults [1, 2]. Current management of type 2 diabetes mellitus involves lifestyle modification: increasing physical activity, improving diet, reaching a healthy body weight and stopping smoking, all monitored by regular screening [2]. If lifestyle modification does not result in sufficiently decreased blood glucose levels, medication may be prescribed, of which there are a range of treatment options available [2]. The complication with type 2 diabetes mellitus is the long latency period, often lasting several years, during which time the individual is often asymptomatic and unaware of their condition [1, 2]. This prolonged asymptomatic state results in long-term damage to the body’s organs that leads to negative health outcomes including pregnancy complications, oral health problems, disabilities such as blindness, reduced wound healing, foot disease that may require amputation, stroke, heart and kidney disease and death [1–3].

### Description of the intervention

There are many types of screening interventions and strategies that may be used to detect disease in a population often classified as mass, opportunistic and targeted strategies—as presented in Table 1 [2, 6]. This systematic review will use these classifications, but if additional strategies are noted, these too will be included.

**Table 1:** Screening strategies applied to detect diabetes

Mass	Screening of an entire apparently healthy population regardless of risk factors
Opportunistic	Screening of individuals, who may or may not be considered at-risk for diabetes, when presenting for any reason to the health system or other opportunistic interaction (e.g. HIV testing drive)
Targeted	Seeking out and screening individuals from a population who are considered at-risk of developing diabetes (e.g. obese, older age)

The biochemical tests commonly used are fasting plasma glucose (FPG), oral glucose tolerance test (OGTT) and detection of glycated haemoglobin A1C (HbA1c) although there are also urine glucose tests available or random blood glucose tests [2, 6]. In addition, there are a number of risk scores [7, 8], including the Finnish Diabetes Risk Score (FINDRISC) [9] and the American Diabetes Association’s risk test [10]; however, these are not commonly used as stand-alone screening tools. Classification of patients post testing can be termed as in the normal range or as having diabetes, impaired glucose tolerance (IGT) or impaired fasting glucose (IFG) (as presented in Table 2) [1, 3].

**Table 2:** WHO recommended ranges used to classify patients according to blood glucose levels [3]

Diabetes	
Fasting plasma glucose	≥ 7.0 mmol/L (126 mg/dl) OR
2-h plasma glucose*	≥ 11.1 mmol/L (200 mg/dl) OR
HbA1c	≥ 6.5%
Impaired glucose tolerance (IGT)	
Fasting plasma glucose	< 7.0 mmol/L (126 mg/dl) AND
2-h plasma glucose*	≥ 7.8 and < 11.1 mmol/L (140 mg/dl and 200 mg/dl)
Impaired fasting glucose (IFG)	
Fasting plasma glucose	6.1 to 6.9 mmol/L (110 mg/dl to 125 mg/dl) AND (if measured)
2-h plasma glucose*	< 7.8 mmol/L (140 mg/dl)

*Venous plasma glucose 2 h after ingestion of 75 g oral glucose load

### How the intervention might work

**Fig. 1:**
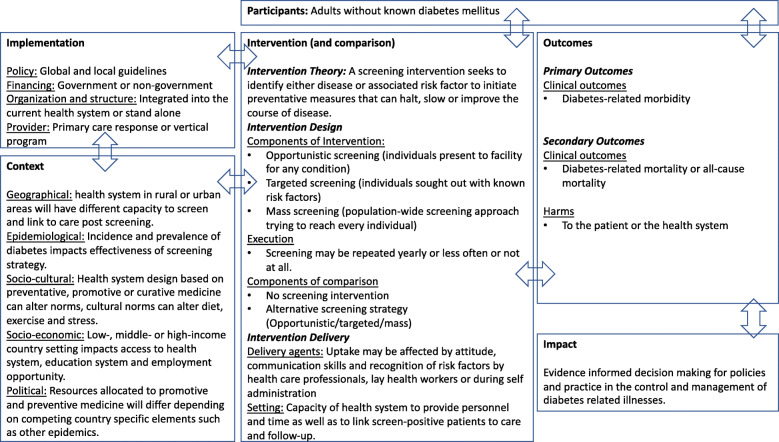
Logic model describing the interactions between screening for diabetes, implementation, context, participants, outcomes and impact

The theory behind screening for type 2 diabetes mellitus is to identify either disease or associated risk factors to initiate preventative measures that can halt, slow or improve the course of disease [11]. Therefore, the earlier the disease is detected, especially where there is high risk of disease, theoretically, the better the expected outcomes. The logic model in Fig. 1 describes a complex system in which the intervention interacts with participants, context, implementation and how these affect the outcomes and the impact of this research [12].

### Why it is important to do this review

Guidelines for screening interventions for type 2 diabetes mellitus, such as those released by the UK National Screening Committee [13], the American Diabetes Association [2] or the Society for Endocrinology, Metabolism and Diabetes of South Africa [14], are based on expert opinion and local practice rather than synthesised evidence. This is because there is limited information to provide evidence about best practice for screening interventions for type 2 diabetes mellitus and even less evidence in low- and middle-income countries [15]. A recently published Cochrane review assessed the effects of any type of screening compared with no screening for type 2 diabetes [16] and found only one trial, the ADDITION-Cambridge trial [17], that met their inclusion criteria. The ADDITION-Cambridge trial consisted of 20,184 participants aged 40–69 years from general practices in England who were at risk for diabetes but had no known diabetes. These participants were randomised to screening versus no screening arms, and followed up for a median of 9.6 years (November 2001 to November 2011). The review found moderate certainty evidence that screening for diabetes probably makes little or no difference to all-cause mortality and low certainty evidence that it may make little or no difference to diabetes-related mortality. However, because the review only included one trial, firm conclusions about early diabetes screening on health outcomes cannot be drawn. In consultation with the authors of the unpublished Cochrane review and considering the public health importance of screening and the potential impact on large populations, we propose to assess evidence from non-randomised intervention study designs. The questions for the systematic review will include the following: Does screening for type 2 diabetes mellitus reduce morbidity and/or mortality? Does a particular screening strategy result in a greater reduction of morbidity and/or mortality as compared to another screening strategy? Does screening for type 2 diabetes mellitus result in harms to participants or the health system?

### Objectives

#### Primary objective

To assess the effectiveness of targeted, opportunistic or mass screening for type 2 diabetes mellitus on reduction of diabetes-associated morbidity in adults

#### Secondary objectives

To assess the effectiveness of targeted, opportunistic or mass screening for type 2 diabetes mellitus on reduction of mortality (all cause as well as diabetes-associated) in adults

To assess the harms of targeted, opportunistic or mass screening for type 2 diabetes mellitus in adults

## Methods

The present protocol has been registered within the PROSPERO database (CRD42020147439). This manuscript is being reported in accordance with the reporting guidance provided in the Preferred Reporting Items for Systematic Reviews and Meta-Analyses Protocols (PRISMA-P) statement [18] (see checklist in Additional file 1).

### Study and source eligibility

#### Types of studies

As existing reviews have found limited randomised evidence addressing this question [15, 19, 20], we will focus on non-randomised intervention studies (NRIS). We will employ the Cochrane EPOC criteria [21], and NRIS of interest will include non-randomised trials, controlled before-after studies, interrupted time-series study, repeated measures study and concurrently controlled prospective cohort study. The difficulty associated with labelling NRIS is well-documented in the literature; several of these designs, for example, have been used interchangeably; we will make use of the EPOC definitions and flow diagram to assist in study design identification (Appendix 1):

- Non-randomised trial (NRT) is a study design in which individual participants, or clusters of participants, are allocated to intervention or comparator in a quasi-random or non-random manner. If there is an allocation rule, it is often by, for example, alternation, day of the week, odd/even hospital, or identification number.
- Controlled before-after (CBA) is a study design that estimates intervention effectiveness by comparing pre- and post-intervention outcomes in individuals or clusters that receive the intervention and those that do not.
- Interrupted time series (ITS) studies design uses multiple observations from individuals or clusters pre-intervention to establish the pre-existing outcome trend; intervention effectiveness is then estimated by measuring post-intervention changes in the expected outcome trend associated with the introduction of an intervention (the ‘interruption’). An ITS study can identify both immediate and long-term changes associated with the intervention. The interrupted time-series studies will be required to have a clearly defined point in time when the intervention occurred and a minimum of 3 time points before and 3 time points after the intervention [21].
- A repeated measures (RM) study is an interrupted time-series study but where the outcomes of interest are measured in the same participants at each point in time.
- Concurrently controlled prospective cohort study (PCS) is where subjects are identified prospectively as having received an intervention or comparator and are then followed over time. The allocation rule is often in relation to organizational factors such as ward, clinic, doctor or provider organisation. Control arms should be contemporaneous, we will not include retrospective control arms.

### ‘PICO’ eligibility

#### Types of participants

We will include adults aged 18 years and older without documented diabetes mellitus or pregnancy.

#### Types of interventions

We will include studies comparing one of the screening strategies, targeted, opportunistic or mass screening interventions for the detection of type 2 diabetes mellitus, against no screening or another of the screening strategies (Table 1). There will be a 6-month minimum follow-up time required for the primary clinical outcome of morbidity.

#### Types of outcome measures

##### Primary outcomes

*Clinical outcomes*

- Diabetes-related morbidity defined as study-reported microvascular complications (diabetic retinopathy, diabetic nephropathy, diabetic neuropathy) or macrovascular complications (non-fatal myocardial infarction, peripheral arterial disease, non-fatal stroke) and measured from 6 months after screening

##### Secondary outcomes

*Clinical outcomes*

- Mortality (all-cause and diabetes-related) defined as death due to any-cause including diabetes or other cardiovascular causes (including acute myocardial infarction, ischemic heart disease, stroke or any cardiovascular disorder that lead to death) and measured at any time after screening

*Harms of diabetes screening*

Harms to patients is defined as event/s reported in the study at any time after screening.

- Psychological harms such as anxiety or stigma that impacts on quality of life due to a false-positive test
- Number of days of work lost
- Side-effects from treatment
- Loss of health insurance benefits

Harms to health care system is defined as event/s reported in the study at any time after screening.

- False-positive test resulting in human, physical and financial resource allocation to patients who are not in need
- Overdiagnosis may lead to over-extension of human, physical and financial resources for patients who end up in prolonged treatment and engagement with the health system even if they never develop disease

The rationale for prioritisation of outcomes: Primary outcome serves to inform whether screening alters the course of disease as assumed per screening theory [11] and depicted in Fig. 1. Secondary outcome of mortality contributes to the current data outlining no reduction in mortality following screening intervention [19] while also assessing harms that may arise from screening intervention [3] and therefore contribute to evidence to substantiate policy and practice recommendations.

### Search methods for identification of studies

#### Electronic searches

The University of Cape Town Health Sciences Reference Librarian (MS) assisted the first author (HM) in developing the search strategy and will provide advice and guidance in conducting the searches for the review.

##### Electronic Database Search (from inception onwards)

- PubMed (MEDLINE)
- Scopus (includes majority of EMBASE contents)
- Web of Science Platform (Web of Science Core Collection, Biological Abstracts, SciELO Citation Index)
- Academic Search Premier (on the EBSCOhost platform)
- CINAHL (on the EBSCOhost platform)
- Health Source Nursing Academic (on the EBSCOhost platform)

A draft search strategy for PubMed/MEDLINE, based on the original search strategy utilised by the Cochrane Review team and revised by an information specialist, is provided in Appendix 2 (see Appendix 2). We will include all studies regardless of publication status; however, we will only include English language studies. We are aware that this decision may lead to language bias [22], but due to capacity and resource limitation of the systematic review team, we are restricted to English only. We will search all databases from inception to the date of search. The search syntax will first be tested and optimised in PubMed. We will thereafter replicate the searches in the other databases adapting search syntax as necessary for those databases.

##### Grey literature search

We will conduct a grey literature search to identify studies not indexed in the databases listed above.

- OpenGrey (multidisciplinary European database, covering science, technology, biomedical science, economics, social science and humanities)
- Conference abstracts from The American Diabetes Association (ADA), the European Association for the Study of Diabetes (EASD) meeting and Diabetologia will be used to track down full text articles.
- National Institute for Health Research Economic Evaluation Database (NHS EED)
- Cost-Effectiveness Analysis Registry (CEA) (www.healtheconomics)

We will search key references, such as systematic reviews, by cross-checking reference lists for additional potentially eligible primary studies [23]. We will also contact experts in the field to check if we have missed any relevant studies. We may contact authors of included studies to clarify reported published information and to seek unpublished data.

### Methods for screening search results

#### Screening methods

We will collate and transfer search results to the Rayyan screening software [24] and remove duplicate records. At least two review authors will independently screen titles and abstracts of every record retrieved. Outcome measures will not be used to exclude studies during title and abstract screening. The potentially eligible records will be retrieved for full text screening. The two review authors will independently review full text records for compliance of studies with eligibility criteria of the review. A decision tree based on the eligibility criteria will be used to assist in decision making for exclusion of studies (see Appendix 3). Two review authors will resolve any disagreements through discussion or, if required, will consult a third review author. A study must meet all inclusion criteria to be included. We will list excluded studies at the full text screening stage in the ‘Characteristics of excluded studies’ table. We will collate multiple reports of the same study so that each study rather than each report is the unit of interest in the review. We will provide any information we can obtain about ongoing studies. We will record the selection process in sufficient detail to complete a PRISMA flow diagram [25].

### Data collection and analysis

#### Data extraction

We will use a standard data extraction form in Microsoft Excel to capture study characteristics and outcome data [22, 26]; we will pilot the form on at least one eligible study. One review author will extract the following study characteristics from the included studies, and an independent review author will check the extraction:

1. Source: study ID (created by review author), review author ID (created by review author), citation and contact details
2. Eligibility: confirm eligibility for review, reason for exclusion
3. Methods: study design, number of study centres and location, study setting, withdrawals, date of study, follow-up, confounding factors considered, and the methods used to control for confounding, aspects of risk of bias specific for NRIS (see “Assessment of risk of bias in included studies” below), how missing data was handled
4. Participants: number, mean/median age, age range, gender, severity of condition, diagnostic criteria, inclusion criteria, exclusion criteria, screening criteria, diagnostic criteria, presence of known risk factors for type 2 diabetes mellitus (obesity, family history), co-morbidity (hypertension, dyslipidaemia), socio-demographics
5. Interventions: intervention components, comparison, fidelity assessment using the Template for Intervention Description and Replication (TIDieR) as a guide [27]
6. Outcomes: primary and secondary outcomes specified above in the section “Types of outcome measures”.
7. Miscellaneous: funding source, notable conflicts of interest of study authors, ethical approval, key conclusions of the study authors, miscellaneous comments from the study authors, references to other relevant studies, correspondence required, miscellaneous comments by the review authors.

One review author will extract outcome data from included studies, and an independent review author will check extracted data. We will note in the ‘Characteristics of included studies’ table if outcome data were reported in an unusable way. We will resolve disagreements by consensus or by involving a third review author.

#### Assessment of risk of bias in included studies

Two review authors will independently assess risk of bias for each study using the ROBINS-I tool [28]. Any disagreement will be resolved by discussion or by involving a third review author.

We will assess the risk of bias according to the following domains:

1. Pre-intervention: bias due to confounding
2. Pre-intervention: bias in selection of participants into the study
3. At intervention: bias in classification of interventions
4. Post-intervention: bias due to deviations from intended interventions
5. Post-intervention: bias due to missing data
6. Post-intervention: bias in measurement of outcomes
7. Post-intervention: bias in selection of the reported result

We will judge each potential source of bias as low risk, moderate risk, serious risk, critical risk of bias or no information. We will summarise the ‘Risk of bias’ judgements across different studies for each of the domains listed. We will consider blinding separately for different key outcomes where necessary (e.g. for unblinded outcome assessment, risk of bias for all-cause mortality may be very different than for a patient reported pain scale). Where information on risk of bias relates to unpublished data or correspondence with a trialist, we will note this in the ‘Risk of bias’ table. We will not exclude studies on the grounds of their risk of bias but will clearly report the risk of bias when presenting the results of the studies. When considering treatment effects, we will take into account the risk of bias for the studies that contribute to that outcome. We will conduct the review according to this published protocol and report any deviations from it in the ‘Differences between protocol and review’ section of the systematic review.

### Dealing with missing data

Authors will be contacted, and missing data will be requested. If only returned in part and data can be logically imputed, such as standard errors, this will occur. All missing data will be clearly reported in the data extraction forms and risk of bias table and as such be assessed in the sensitivity analysis.

### Data management

EndNote X9 and Microsoft Excel will be used for data management. If there is a conflict between data reported across multiple sources for a single study (e.g. between a published article and a trial registry record), we will report the data from the first peer-reviewed published article.

### Data synthesis

#### Preparation for data synthesis

In preparation for synthesis (either meta-analyses or synthesis without meta-analysis), we will assess how much data are available for each of our objectives by creating a table to compare the PICO elements and the study design features as well as the extracted numerical data for the compilation of a meta-analysis.

### Measures of treatment effect

We will estimate the effect of the intervention using risk ratio for dichotomous data, and mean difference or standardised mean difference for continuous data. Time to event outcomes will be reported as hazard ratios. If other effect estimates are provided, we will convert between estimates where possible. Measures of precision will be 95% confidence intervals. We will ensure that an increase in scores for continuous outcomes can be interpreted in the same way for each outcome, explain the direction to the reader, and report where the directions were reversed if this was necessary. Interrupted time series data will be analysed and, if required, a statistical comparison of time trends before and after the intervention will be performed. For ITS studies, the guideline as outlined in Analysis in EPOC reviews will be followed with assistance of a statistician to ensure integrity of analysis [29].

### Unit of analysis issues

To avoid unit of analysis errors we will consider the unit used to cluster the intervention (such as a ward, clinic, doctor or provider organisation) or in the case of repeated measures that there will be multiple observations for the same outcome. For instance, multiple screening intervention events per participant may occur over time that may cause a unit-of-analysis error. In order to calculate the confidence intervals, the participants per treatment group rather than the number of intervention attempts will be used [22]. Multiple intervention groups could create unit-of-analysis issues especially if different screening interventions are compared against no screening intervention and use the same participants with no screening intervention in both comparisons [22]. If there is more than one comparison in the study design, we will combine groups into a single pairwise comparison. If there is a unit of analysis error in the reported analysis for a study and there is insufficient information to reanalyse the results, the study authors will be contacted to obtain necessary data. If these data are not available, we will not report confidence intervals or *p* values for which there is a unit of analysis error [30].

### Quantitative synthesis

We will undertake meta-analyses only where this is meaningful, i.e. if the interventions, participants and the underlying clinical question are similar enough for pooling to make sense. If feasible and appropriate, outcome data from primary studies will be used to perform random effects meta-analyses. Since heterogeneity is expected a priori, we will estimate the pooled treatment effect estimates and its 95% confidence interval using the random effects model. The random effects model assumes that the effect estimates follow a normal distribution, considering both within-study and between-study variation.

### Assessment of heterogeneity

Forest plots will be used to visualise the extent of heterogeneity among studies. We will quantify statistical heterogeneity by estimating the variance between studies using *I*^2^ statistic. The *I*^2^ is the proportion of variation in effect estimates that is due to genuine variation rather than sampling (random) error. *I*^2^ ranges between 0 and 100% (with values of 0–25% and 75–100% taken to indicate low and considerable heterogeneity, respectively) [22]. We will also calculate the chi-squared test where a *p* value < 0.1 indicates statistically significant heterogeneity.

### Assessment of publication bias

If we include more than 10 studies investigating a particular outcome, we will use a funnel plot to explore possible publication bias, interpreting the results with caution [31].

### Subgroup analysis and investigation of heterogeneity

We expect the following population characteristics may introduce clinical heterogeneity: age, sex, socio-economic status [6].

We expect the following contexts may introduce health system heterogeneity: study setting of rural or urban or in a low-middle income country or a high-income country (as defined by the World Bank) [6].

We will use the following outcomes in subgroup analysis:

1. Diabetes-associated morbidity
2. Mortality (all-cause and diabetes-associated)
3. Harms

### Sensitivity analysis

We may conduct a sensitivity analysis to explore the influence of various factors on the effect size of the primary outcomes of the review only. We will stratify studies according to:

1. Restricting the analysis to published studies.
2. Restricting the analysis to studies with a low risk of bias, as specified in “Assessment of risk of bias in included studies”
3. Imputing missing data.

Any post hoc sensitivity analyses that may arise during the review process will be justified in the final report.

### Assessment of certainty of evidence using the GRADE approach

Two review authors will independently assess the certainty of the evidence (high, moderate, low and very low) for each outcome using the five GRADE considerations for downgrading the certainty of evidence (risk of bias, consistency of effect, imprecision, indirectness, and publication bias) and the three criteria for upgrading the certainty of evidence (large effect, dose response and residual confounding opposing the observed effect) [32]. We will use the GRADEpro software GDT [33] to create the ‘Summary of findings’ tables for the main intervention comparisons and include the following outcomes: diabetes-associated morbidity, mortality (all-cause and diabetes-associated), harms (see Appendix 4 for SoF). We will resolve disagreements on certainty ratings by discussion and provide justification for decisions to down- or upgrade the ratings using footnotes in the SoF table and make comments to aid readers’ understanding of the review where necessary. We will use plain language statements to report these findings in the review [34]. The SoF tables will be used to draw conclusions about the certainty of the evidence within the text of the review. If during the review process, we become aware of an important outcome that we failed to list in our planned ‘SoF’ tables, we will include the relevant outcome and explain the reasons for this is the section ‘Differences between protocol and review’.

## Discussion

Systematic reviews of screening for type 2 diabetes have found no evidence that this intervention saves lives [15, 19, 20]; therefore, this review will primarily focus on the impact of screening on the reduction of diabetes-associated morbidities. The impact of this review is synthesised data for the provision of evidence-based decision-making for informing policy and practice around screening strategies for type 2 diabetes mellitus. Important protocol amendments will be documented and noted in the discussion.

## Limitations

The potential limitations of this review at a study (outcome) level include the following: the potential finding of insufficient studies of similar study design and clinical question to synthesise abstracted study data; the overall completeness and applicability of evidence and quality of evidence especially due to the limitation to non-randomised studies due to the lack of randomised studies and therefore the lower quality of evidence; the limitation to English studies only and therefore the potential to miss published research; the limitation of not being able to discern between all-cause mortality and diabetes-related mortality and therefore combining this outcome under one mortality outcome. The potential limitation of this review at a systematic review process level includes the potential biases in the review process such as post hoc analysis and focus of outcome objectives.

### Supplementary information

**Additional file 1.** PRISMA Checklist.

## Figures and Tables

**Fig. 2:**
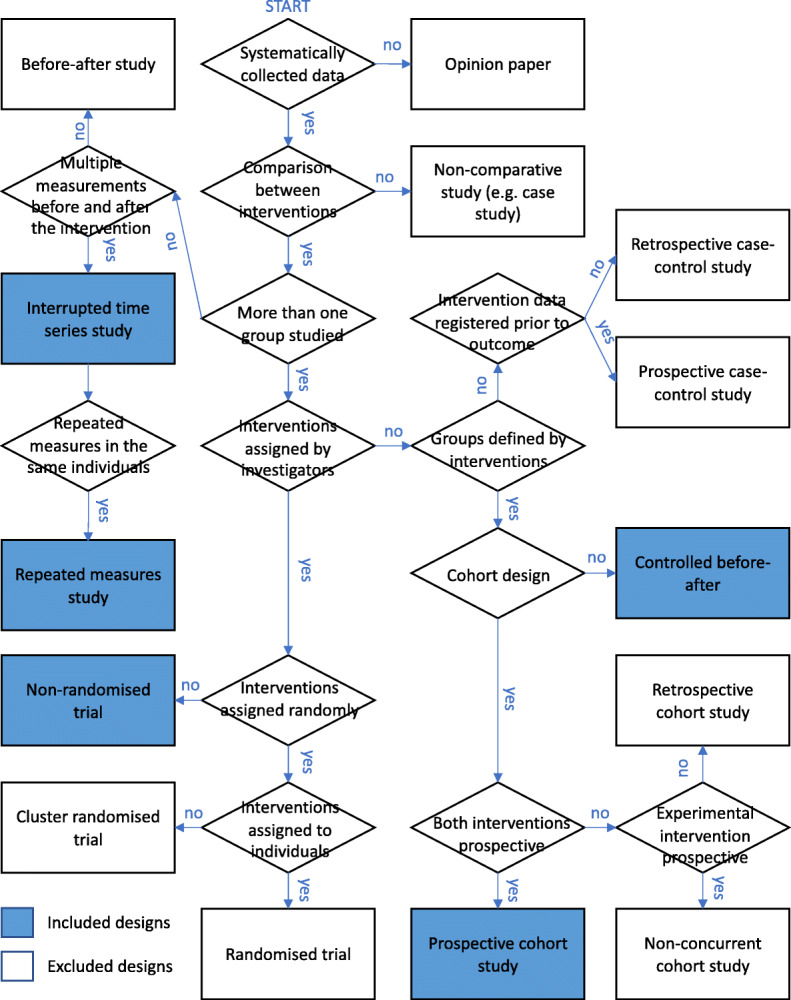
Flow diagram to assist with identifying the type of study (modified from [34])

## Data Availability

Not applicable.

## Appendix 2

### Search Strategy for PubMed:

Set 1: Diabetes Mellitus, Type 2 [MeSH] OR [Text Word field:] Adult onset diabetes OR late onset diabetes OR latent diabetes OR mature onset diabetes OR MODY OR NIDDM OR noninsulin-dependent diabetes OR slow onset diabetes OR stable onset diabetes OR type 2 diabetes OR type II diabetes OR T2DM OR T2D

**Set 2:** Diabetes Insipidus [MeSH] OR [Text Word field:] diabetes insipidus

**Set 3**: 1 NOT 2

**Set 4:** Mass screening [MeSH] OR [Text Word field:] screening

**Set 5:** 3 AND 4

**Set 6:** Animals [MeSH] NOT Humans [MeSH]

**Set 7:** 5 NOT 6

**Set 8:** [All fields:] Trial OR trials OR before-and-after study OR before-and-after studies OR cohort OR comparative study OR comparative studies OR Controlled OR evaluation study OR evaluation studies OR follow-up study OR follow-up studies OR interrupted time series OR longitudinal study OR longitudinal studies OR non-randomised OR non-randomized OR nonrandomised OR nonrandomized OR non randomised OR non randomized OR program evaluation OR programme evaluation OR prospective study OR prospective studies OR quantitative study OR quantitative studies OR quasi experimental OR repeated measures

**Set 9:** 7 AND 8

## Appendix 3

**Table 3:** Provisional eligibility decision tree for full text exclusion

Hierarchy	Exclusion reason	Explanation of reason
1	Duplicate	Record is a duplicate of another study already included in the review
2	Animal study	Study conducted in non-human population
3	Study withdrawn	Study was withdrawn before results became available
4	Ongoing study	Study is ongoing; No study results have been published. Study will be described in ‘Ongoing studies’ section of the review.
5	Wrong intervention	Study does not include screening for type 2 diabetes mellitus
6	Wrong study design	Study is not eligible as per Appendix 1 study designs.
7	Wrong population	Study intervention/ outcomes involve individuals who have type 2 diabetes mellitus or are pregnant
8	Research question is inappropriate	Study is not eligible due to inappropriate research question or objectives that do not address systematic review objectives.
9	Wrong Outcomes	Study does not report outcomes that align with primary or secondary outcome measures.

## Appendix 4

**Table 4:** Provisional summary of findings table

Targeted, opportunistic or mass screening for type 2 diabetes mellitus compared to each other or no screening in children, adolescents and adults.						
**Patients or population**	Children, adolescents and adults without documented diabetes mellitus or pregnancy.					
**Intervention**	Targeted, opportunistic or mass screening for type 2 diabetes mellitus.					
**Comparison**	Other screening (targeted, opportunistic or mass) or no screening for type 2 diabetes mellitus.					
**Outcomes**	Illustrative comparative risks (95% CI)		Relative effect (95% CI)	Number of participants (studies)	Quality of evidence (GRADE)	Comments
	Assumed risk	Corresponding risk				
	With targeted, opportunistic or mass screening	With other screening or without screening				
Diabetes-related morbidity						
Mortality (all-cause and diabetes-related)						
Harms						

## Abbreviations

ADA: American Diabetes Association
CBA: Controlled before-after
CEA: Cost-Effectiveness Analysis Registry
CEBHA+: Collaboration for Evidence-Based Healthcare and Public Health in Africa
EASD: European Association for the Study of Diabetes
EPOC: Effective Practice and Organisation of Care
FPG: Fasting plasma glucose
GRADE: Grading of Recommendations Assessment, Development and Evaluation
HbA1c: Detection of glycated haemoglobin A1C
ITS: Interrupted time series
NHS EED: NHS Economic Evaluation Database
NRT: Non-randomised trial
NRIS: Non-randomised intervention studies
OGTT: Oral glucose tolerance test
PCS: Prospective cohort study
PRISMA: Preferred Reporting Items for Systematic Reviews and Meta-Analyses
RM: Repeated measures
SoF: Summary of findings
TIDieR: Template for Intervention Description and Replication
